# Simple Syntheses of New Pegylated Trehalose Derivatives as a Chemical Tool for Potential Evaluation of Cryoprotectant Effects on Cell Membrane

**DOI:** 10.3390/molecules25030497

**Published:** 2020-01-23

**Authors:** Karel Pomeisl, Jan Richter, Martin Golan, Irena Kratochvílová

**Affiliations:** Institute of Physics, Czech Academy of Sciences, Na Slovance 1999/2, 182 21 Praha 8, Czech Republic; pomeisl@fzu.cz (K.P.); jan.richter@joe.cz (J.R.); martindata6@gmail.com (M.G.)

**Keywords:** click-chemistry, pegylation, cryoprotection

## Abstract

In our work, we developed the synthesis of new polyfunctional pegylated trehalose derivatives and evaluated their cryoprotective effect using flow cytometry. We showed that new compounds (modified trehaloses) bound to appropriate extracellular polymeric cryoprotectants could be helpful as a chemical tool for the evaluation of their potential toxic cell membrane influences. Our aim was to form a chemical tool for the evaluation of cryoprotectant cell membrane influences, which are still not easily predicted during the freezing/thawing process. We combined two basic cryoprotectants: polyethyleneglycols (PEGs) and trehalose in the new chemical compounds—pegylated trehalose hybrids. If PEG and trehalose are chemically bound and trehalose is adsorbed on the cell surface PEGs molecules which are, due to the chemical bonding with trehalose, close to the cell surface, can remove the cell surface hydration layer which destabilizes the cell membrane. This was confirmed by the comparison of new material, PEG, trehalose, and their mixture cryoprotective capabilities.

## 1. Introduction

Cryopreservation of cells plays an essential role in many areas [1,2,3,4,5]. The main difficulty in cryopreservation is that ice crystallization, which appears throughout the freezing process, can significantly damage the cells and cause the loss of viability after the cells are thawed [6]. Fortunately, the freezing process is strongly affected by added substances and a multitude of prokaryotic and eukaryotic cells can be recovered from temperatures as low as almost two hundred degrees below the specific freezing point when different cryoprotectants are present. One of the main challenges of cell cryopreservation is influencing the freezing kinetics, specifically the ice nucleation and growth [7]. Crystallization typically starts in the extracellular space. Freezing forms an osmotic imbalance, which results in a flow of water from the inside of cells [8]. In this case, the concentration of ions and other solvents increases beyond physiological concentrations, which results in chemical stress [9,10,11,12]. With ice crystallization, the osmotic stress is increasing and such dehydration damages membranes and organelles [2]. Another big problem during cell freezing is the growth of intracellular ice crystals. At temperature below −80 °C, the remaining highly concentrated solution within and outside the cells turns into a glassy matrix, which is the relatively stable form for long-term preservation. It is, therefore, crucial to develop cryoprotective substances and methods lacking the negative side-effects, but first, it is necessary to deepen the know-how required for their rational design.

In this work, we combined two basic cryoprotectants: polyethyleneglycols (PEGs) and trehalose in the new chemical compounds (polyfunctional pegylated trehalose hybrids). Our aim was to form a chemical tool for the evaluation of cryoprotectant influences on the cell membrane, which are still not easily predicted during the freezing/thawing process. In [13,14,15], it has been shown that the state of the cell membrane (structure, stiffness, raptures in the membrane) correlates with frozen/thawed cell viability [2,12]. Saccharides are expected to stabilize biological molecules [16,17]. Trehalose is able to intercalate into the outer phospholipid layer of the cell membrane and stabilize it by hydrogen bonding to the polar parts of its phospholipids [18,19]. Polyethylene glycol (PEG) is an extracellular cryoprotectant inhibiting ice crystal growth and increasing tonicity of vitrification solutions, which helps to prevent chilling injury [20]. On the other side, high PEG concentrations substantially alter the state of the cell membrane surface [9], e.g., PEGs remove the hydration layer that impedes the close apposition of converging phospholipid bilayers [10,21,22]. PEG interactions with water increase repulsions of lipid polar heads which further results in the destabilization of cell membranes [23].

**Figure 1:**
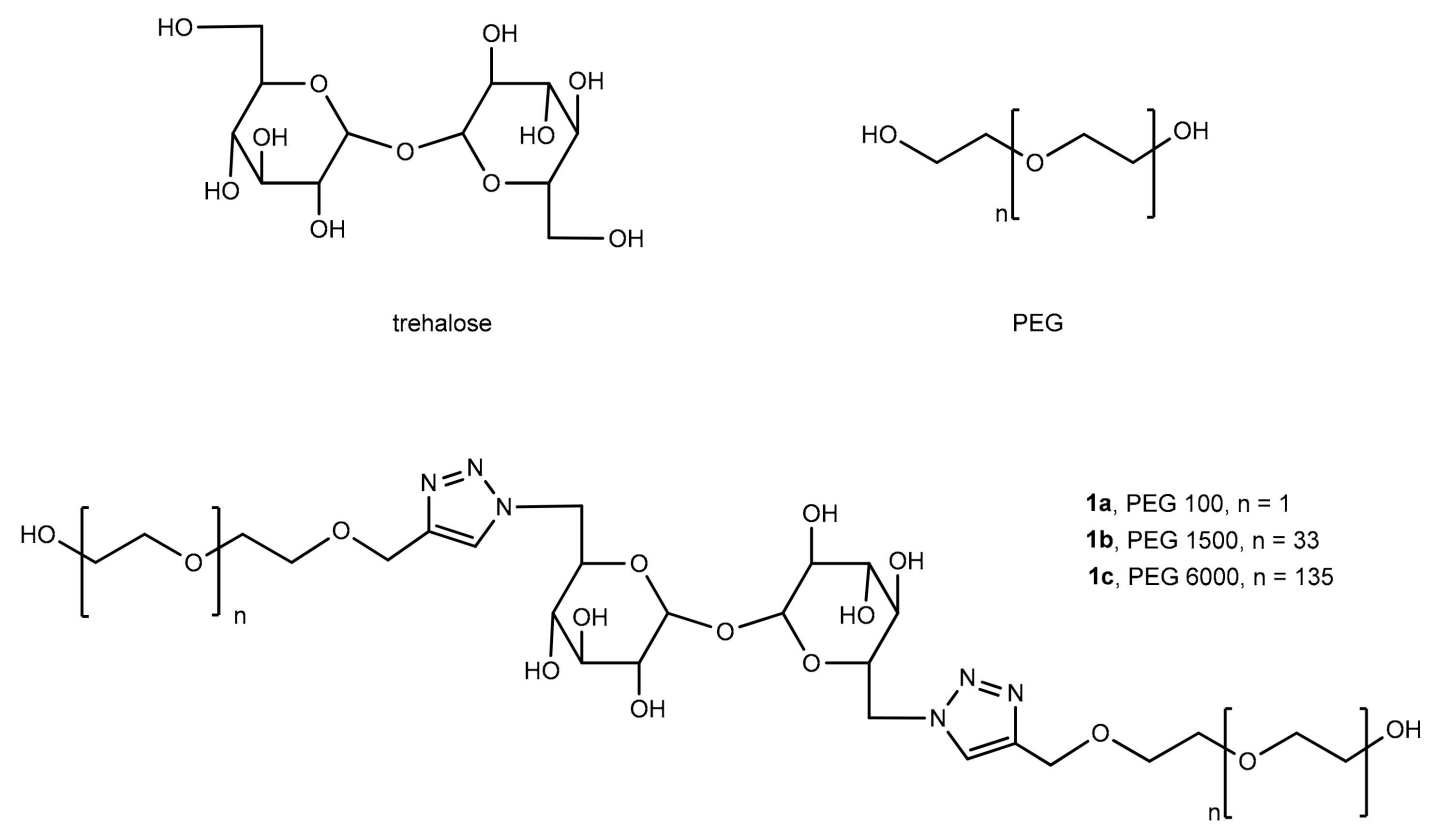
Chemical structures of pegylated trehalose hybrids **1a**–**c** under study.

Synthetically modified trehalose bound to appropriate extracellular polymeric cryoprotectants (PEG) may be helpful as an auxiliary chemical tool for evaluation of their toxic cell membrane influences, which are still not precisely described (see Figure 1). In addition, the functionalization of trehalose may find utility in the field of cryoprotectant design, considering little exploration of syntheses which deal with new chemicals [24].

## 2. Results and Discussion

### 2.1. Development of Synthesis of Pegylated Trehalose Hybrids

**Scheme 1:**
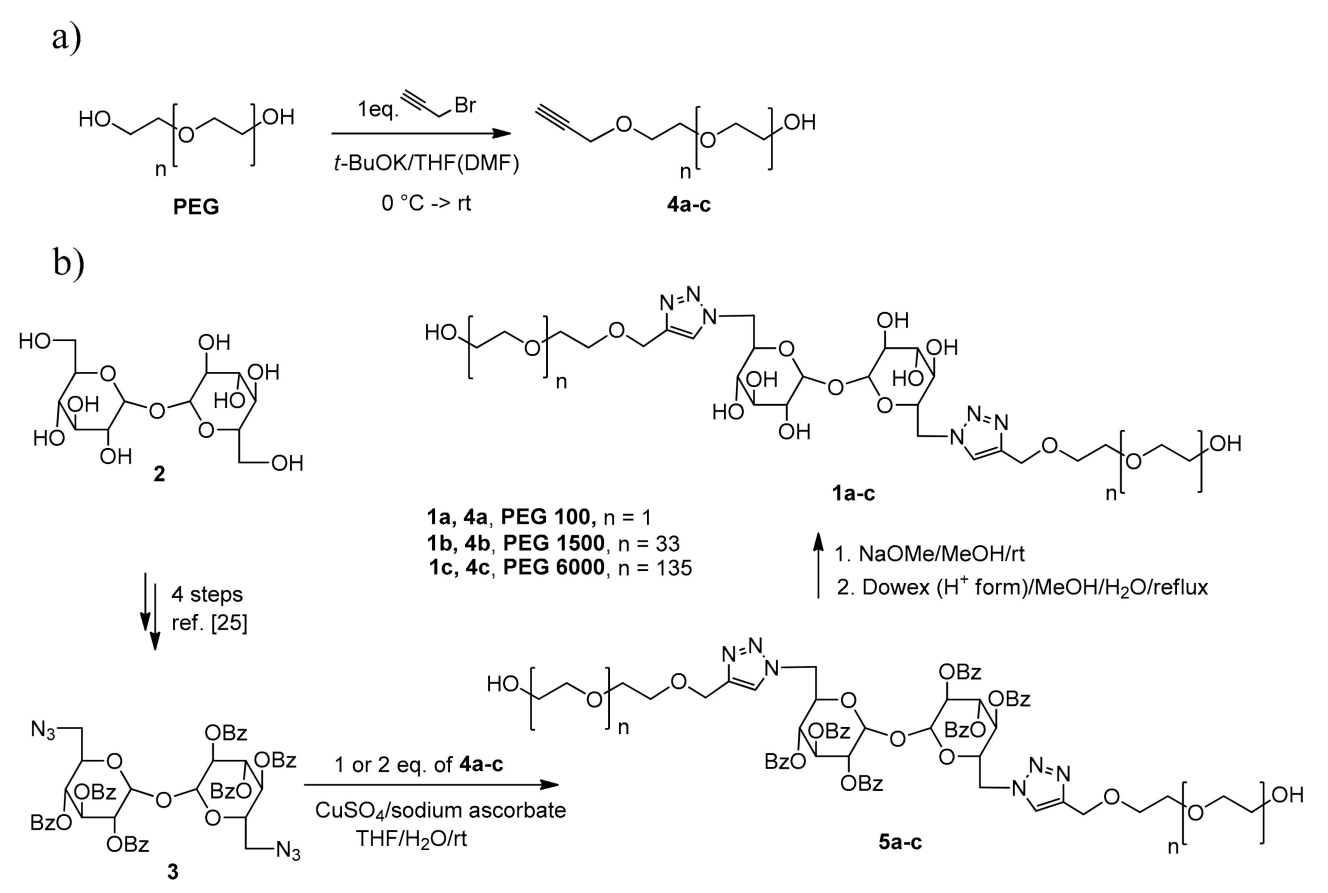
Synthesis of propragylated PEGs **4a–c** (**a**) and pegylated trehalose derivatives **1a–c** (**b**) [25].

The low reactivity, selectivity and specific methods of disaccharide skeleton protection lead us to symmetrical modification at 5′-carbon to activate a potential polymer spacer. The polymer insertion at the 5′-carbon also seems to be more appropriate for the spatial direction of pegylated tentacles while secondary hydroxyl groups are still available for assumed membrane interactions. So, we modified the 5′-hydroxyl group of trehalose by the multi-step synthesis as reported in ref. [25]. Unfortunately, the direct condensation of **2** with PEG 1500 in the presence of various bases and under various reaction conditions did not proceed regarding that no product was observed in the reaction mixtures which was monitored by NMR spectroscopy (see Supplementary Material). Therefore, we described a simple click-condensation of propargylated PEGs 100, 1500 and 6000 with trehalose 5,5′-diazidoderivative **3** (see Scheme 1).

Two important characteristic features make click chemistry so attractive for assembling compounds, reagents, and biomolecules for preclinical and clinical applications. Click reactions are bio-orthogonal—functional groups of neither the reactants nor their products interact with functionalized biomolecules [26,27].

As the cytotoxicity of triazole derivatives was mild [27,28,29], we suggested the appropriate synthetic conditions of this method for the development of new polymeric hybrids **1a**–**c**. The synthetic protocol is based on a good reactivity of the primary hydroxy group at 5′-position of trehalose **3**. Contemporaneously, the secondary hydroxyl substituents are still available for proposed interaction with a cell membrane. For the pegylation, we chose monopropargylated PEG synthons **4a**–**c** which were selectively alkylated of commercial PEGs with the equivalent of propargyl bromide in the presence of potassium *tert*-butyloxide [26]. Diazidoderivative **3** was further coupled with propargyl derivatives **4a**–**c,** whereas copper sulfate in the presence of sodium ascorbate was used as a suitable catalyst for 1,3-dipolar cycloaddition [30]. The corresponding pegylated intermediates **1a**–**c** were obtained in 13–55% preparative yields. Double pegylated chains of pegylated products were confirmed by NMR and mass spectroscopy using ESI or MALDI-TOF (see Materials and Methods). For example, the characteristic chemical shifts of triazol proton H^’^-5 (7.52 ppm) clearly proves the click products **5a**–**c**. This finding also supports the presence of triazol carbon C’-4 at 145.3 ppm together with singlet carbon (C’-5) 123.07 ppm and the change of a number further chemical shifts such as trehalose protons H-6 from 2.08 ppm to 4.18 ppm, H-4 from 5.42 ppm to 5.34 ppm and H-2 from 5.47 ppm to 5.23 ppm in consequence of different electron shielding effect of triazol conjugated system. Regarding the trehalose is containing two symmetric glucose units, the ratio of the integrals of the peaks for the triazol protons H’-5 7.52 ppm and all trehalose signals such as 4.18, 5.23, 5.34, 5.51, 6.19 ppm or polyethyleneglycol protons at 3.72 ppm and 4.73 ppm corresponds the ratio of polymer/sugar 1:1 per glucose unit. The presence of glucose dimer bearing non-reductive glycosidic bond was proved by using Feling’s solution

Based on this characterization of double pegylated products, the reaction conditions were subsequently modified to find any dependence on regioselectivity for the alkylation step. That means the decreasing ratio of **4**/**3** from 2:1 to 1:1 surprisingly leads only to the double pegylated product. The competitive monopegylation of diazidotrehalose was observed by neither TLC nor NMR-monitoring of the reaction mixture. That means no doubled trehalose proton signals or decrease of intensities of triazol or polyethylenglycol signals toward trehalose was found. The analysis of reaction mixture by TLC proved only the presence of non-polar double pegylated products (Rf = 0.45 in dichloromethane/MeOH 15:1-2) which were further characterized after their separation. In the case of the equivalent ratio of **4** to **3**, the low preparative yields of 1 were obtained whereas the conversion of 5,5′-diazidotrehalose did not complete as observed by TLC and NMR-monitoring. These results may indicate that double coupling of strongly reactive 5,5′-diazidotrehalose **3** with propargylated PEGs **4a**–**c** was compensated by a lower conversion of starting 5,5′-diazidotrehalose.

Benzoyl protecting groups were fully removed by treatment of sugars with sodium methoxide overnight to give 36–77% preparative yields.

### 2.2. Determination of Cryoprotective Effect of New Pegylated Trehalose Hybrids

**Figure 2:**
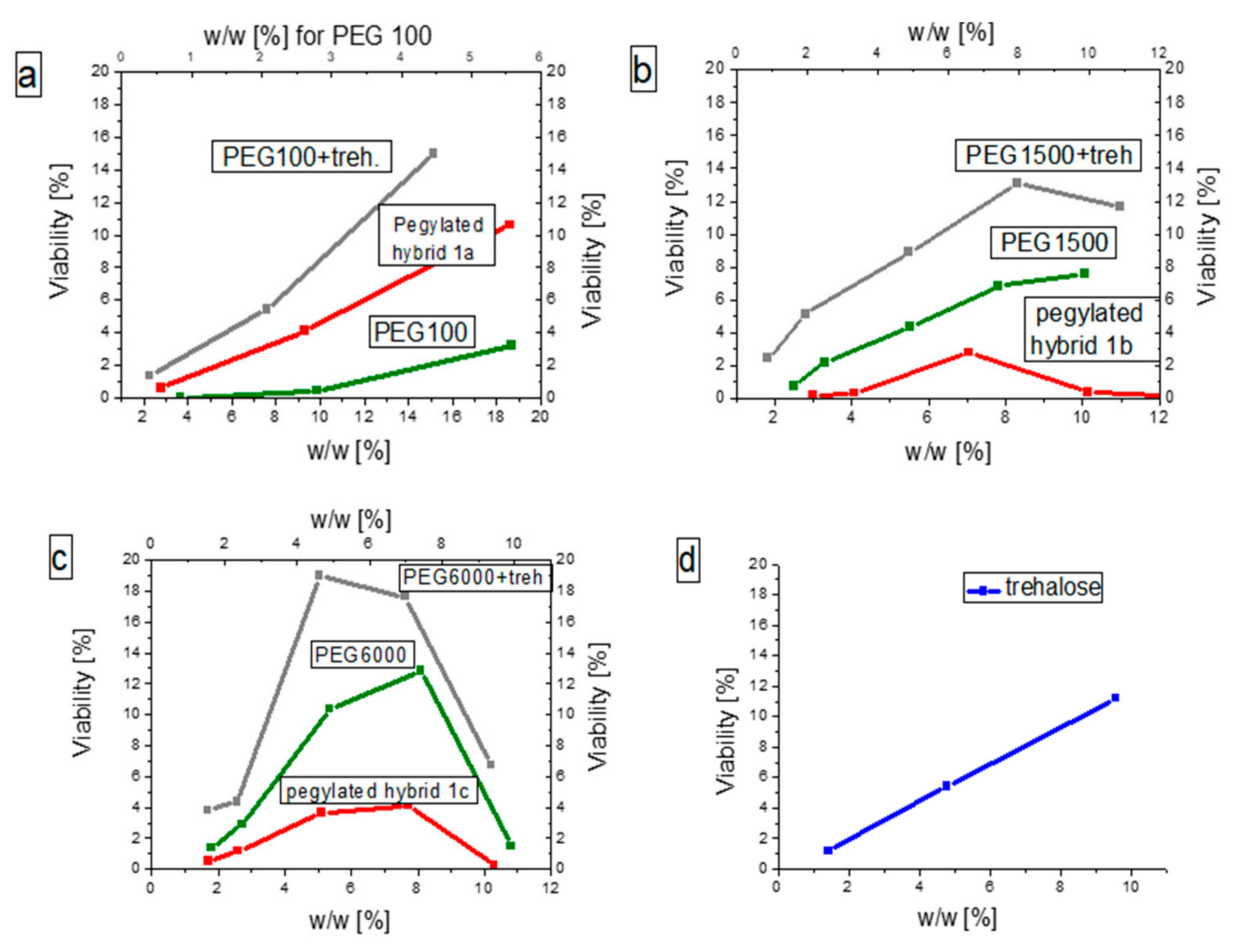
Determination of viability of frozen/thawed human mantle cell lymphoma (Z138-MCL) treated by cryoprotectants at different concentrations (mass fraction *w/w* [%]): (**a**) PEG 100, mixtures of PEG 100 and trehalose, pegylated hybrids **1a**; (**b**) PEG 1500, mixtures of PEG 1500 and trehalose, pegylated hybrids **1b** (**c**) PEG 6000, mixtures of PEG 6000, trehalose and pegylated hybrids **1c**; (**d**) trehalose. Comparison of cell viability was performed by flow cytometry. For each pegylated hybrid, three distinct controls in corresponding stoichiometric concentrations: trehalose, PEG, mixture of trehalose and PEG. The total amount of cells in the samples was 60,000 ± 4000.

**Figure 3:**
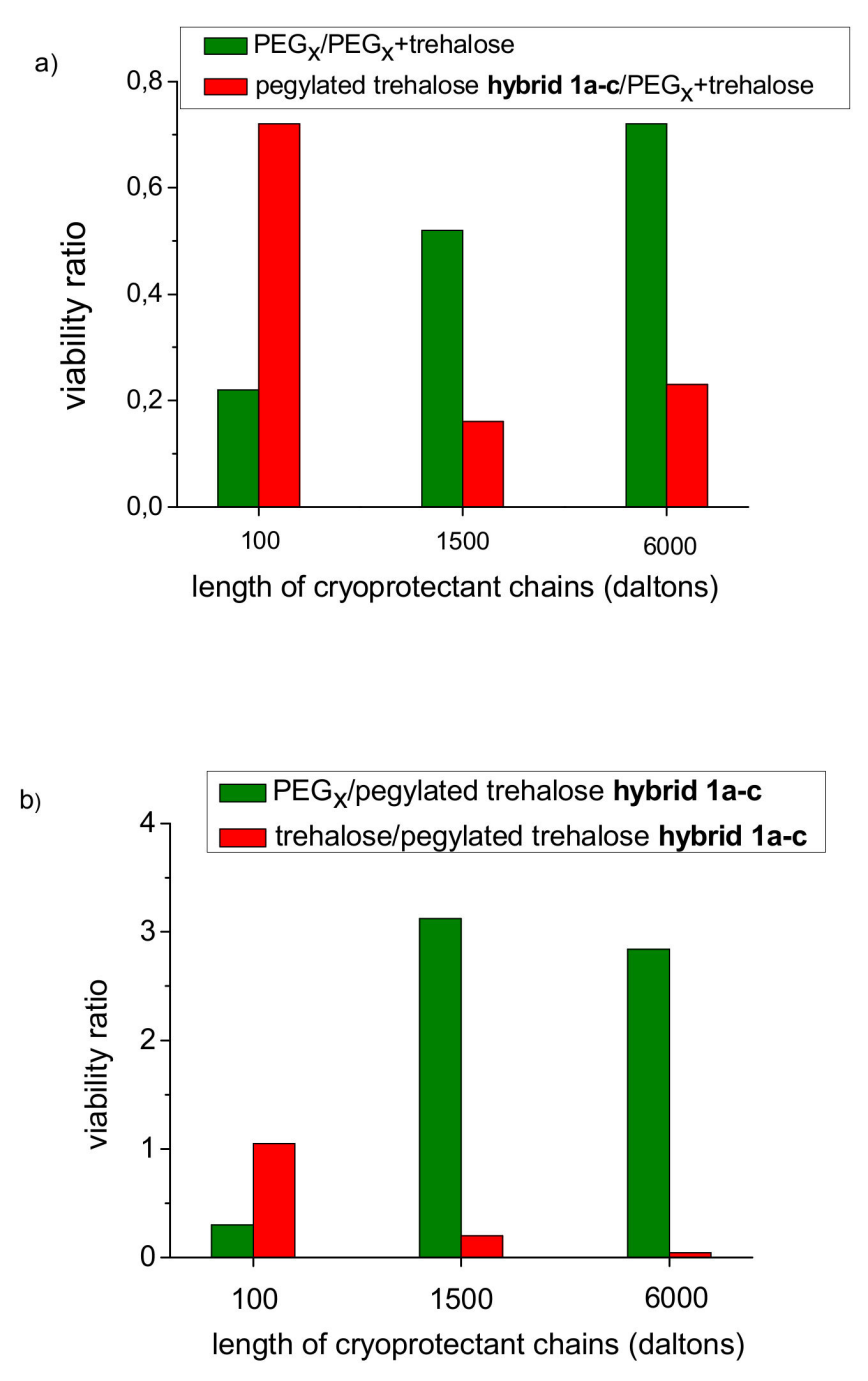
Determination of viability at comparable stoichiometric concentration of cryoprotectants: (**a**) Viability ratio of PEG_x_ (green) and **1a**–**c** (red) treated frozen/thawed cells related to viability of cells treated by a mixture of PEG_x_/trehalose; the efficiency of cryoprotectivity for **1a**–**c** was observed as a potential result of the fact that short pegylated linkage of **1a** prevents a high destabilization of cell membrane; approaching of middle PEG-chains to cell membrane increases the probability for stronger interactions with water resulting in the destabilization of membrane (**b**) Viability ratio of PEG_x_ (green) and trehalose (red) treated frozen/thawed cells related to viability of cells treated by pegylated hybrids **1a**–**c**.

The cryoprotective effects of the products **1a–c** were tested using flow cytometry (see Figure 2 and Figure 3). Flow cytometry is a widely used method based on high-throughput evaluation of biological/physical parameters of fluorescent-labeled cells in suspension. One of its many uses is the determination of the percentage of living and/or dead cells within a sample based on the presence or absence of fluorescence markers. In this study, we used Annexin V for the determination of apoptotic cells and propidium iodide (PI) for the detection of necrotic cells. The percentage of viable cells presented here is the ratio of cells lacking both these markers to all cells in the sample. For investigation, the standard freezing/thawing protocol was used and the viability was evaluated on the human mantle cell lymphoma Z138-MCL. Flow cytometry is based on high-throughput analysis of thousands of individual cells (data points) in one sample, so multiple simultaneous measurements (doublets, triplets, etc.) are not commonly used in flow cytometry evaluation. The viability was measured immediately after a freeze/thaw cycle. The general viability of cancer cells is not the decisive point compared to the much more significant impact of freezing/thawing. The significance of our compounds is not in their cryoprotective capacity but rather in the ability to explain some of the freezing/thawing-related processes.

We determined the cryoprotective effects of solutions containing various concentrations of pegylated hybrids **1a**–**c** respectively (Figure 2). For each pegylated hybrid, three distinct controls of cryoprotectivity in corresponding stoichiometric concentrations were measured for trehalose, PEGs, and a mixture of PEG_x_/trehalose. When PEG control was used as cryoprotectant the effective viability values of cryopreserved cells were higher for longer PEG chains (viability of PEG 6000 treated cells > viability of PEG 1500 treated cells > viability of PEG 100 treated cells). In contrast, long (**1b, 1c**) hybrids had less cryoprotective effect compared to corresponding long (1500, 6000) PEGs. However, it is also evident that the cryoprotective activities of pegylated hybrids are supported by trehalose fragment which may also confirm the proposed trehalose interaction with a complex membrane directly on living cells.

**Figure 4:**
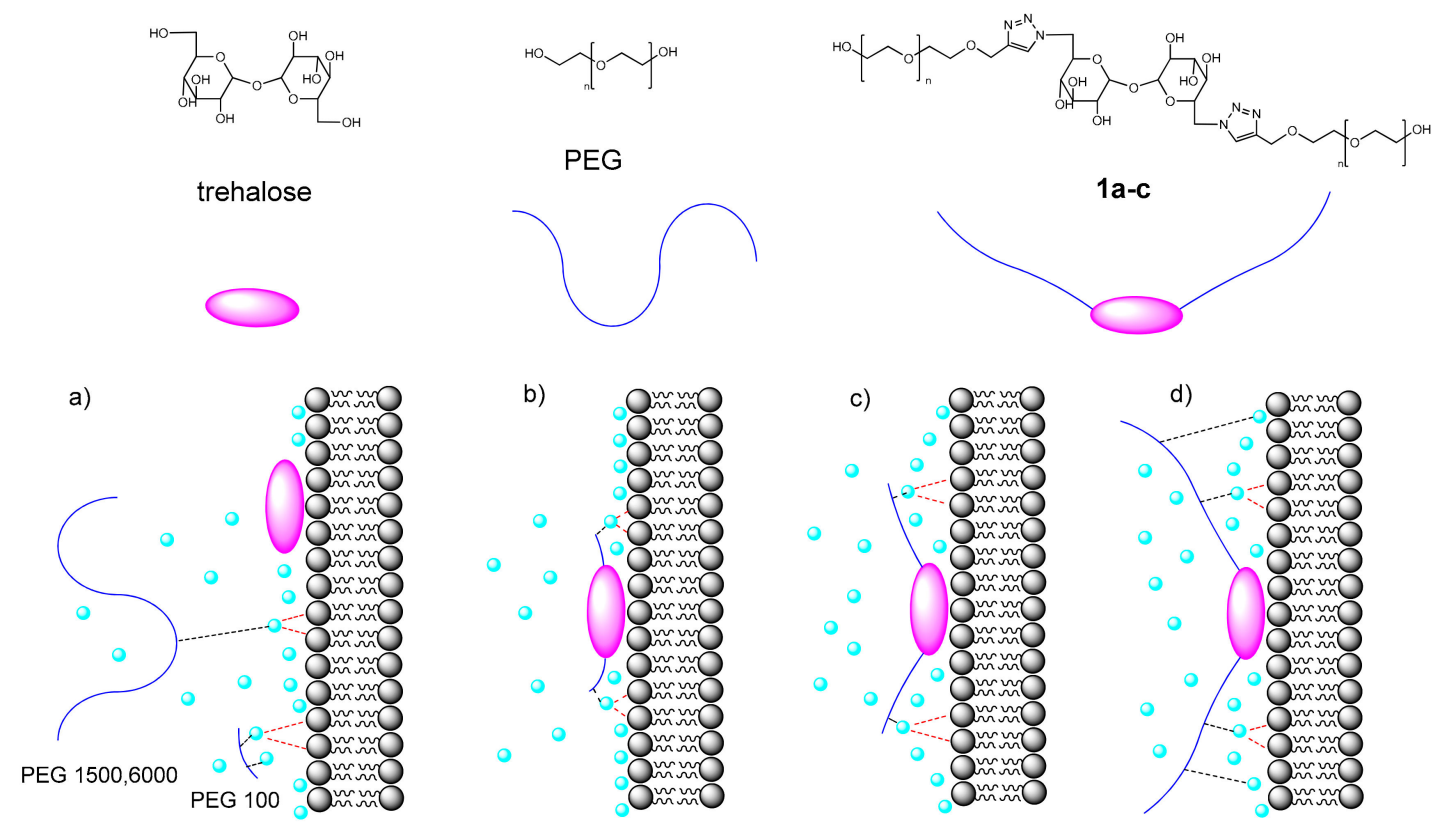
Proposal of chelate effect: (**a**) The proposed destabilization of polar lipid heads through hydrogen interactions of PEGs with membrane water [21,32] the interaction of polymer chains of PEG 1500 and PEG 6000 to the water which bound to membranes is weaker in comparison with short PEG 100. The assumed stabilizing effect of cell membranes by trehalose displacement with water; (**b**) The short pegylated linkage of **1a** prevents a high destabilization of cell membrane; (**c**) Approaching middle PEG-chains to cell membrane increases the probability for stronger hydrogen interactions with water resulting in the destabilization of membrane at the concentration comparable with pegylated hybrids **1b**; (**d**) the more flexible pegylated hybrid **1c** may partially deflect its PEG-tentacles from the membrane surface.

A decrease of cell survival for new materials under study in order of their pegylated chains was observed: cryoprotective effect of pegylated hybrid **1a** (containing PEG 100) > cryoprotective effect of pegylated hybrid **1c** (containing PEG 6000) > cryoprotective effect of pegylated hybrid **1b** (containing PEG 1500). In addition, we observed various changes of viability for pegylated hybrids towards a cryoprotective mixture of PEG and trehalose as a control. While the cryoprotective effect of PEG chains gradually increased depending on their growing chain length, longer/more rigid pegylated hybrids exhibited considerably lowered efficiency to a minimum value for the middle chain compound (Figure 3). This phenomenon suggests the more flexible structure of **1c** in which the significantly longer pegylated tentacles may be partially deflected from the membrane surface compared with **1b** (see Figure 4).

The observed differences in viability indicate that the rigidity of a covalently bound polymer to sugar can be a limiting factor of new materials application as cryoprotectants. A considerably higher cryoprotective efficiency of PEG substrates (compared to pegylated trehalose hybrids) clearly shows the effect of linking trehalose to long-chain PEGs on cryoprotective efficiency. On the other hand, compound **1a** containing PEG 100 exhibited a comparable cryoprotective efficiency with trehalose whereas its higher cryoprotective efficiency compared to the 100 PEG chain suggests a marginal role of a short PEG chains membrane destabilizing interactions with water on the cell surface (see Figure 2 and Figure 3).

The loss of viability of new products related to a mixture of PEG and trehalose high viability could be preliminarily explained by potential chelate effect (Figure 4) [31]. The increase of hydrogen interactions of PEG chains bonded to trehalose which replaces water molecules on the cell surface may deepen the destabilization of the cell membranes. Compared to pegylated hybrid **1a** (containing PEG 100) longer pegylated chains can be more flexible to form stronger hydrogen interactions with water.

## 3. Materials and Methods

All starting materials and reagents were purchased from Sigma-Aldrich. All solvents used for the synthesis were “p.a.” grade. The commercial PEG polymers were purchased from Sigma-Aldrich. ^1^H and ^13^C NMR spectra were recorded on a Varian Gemini 300 HC instrument—deuteriochloroform (CDCl_3_) and hexadeuteriodimethyl sulfoxide (DMSO-*d*_6_) were used as solvents and signals of the solvent served as an internal standard. Chemical shifts (δ) are given in ppm and *J* values are given in Hz. ^13^C NMR signals were identified by APT experiments. Mass spectra were measured by ESI technique on LCQ Fleet or LTQ Orbitrap XL (Thermo Fisher Scientific, Waltham, MA, USA) and MALDI technique on UltrafleXtreme™ MALDI-TOF/TOF mass spectrometer (Bruker Daltonics, Bremen, Germany). General procedure for materials synthesis—propargylated PEGs **4a**–**c**, pegylated trehaloses **1a**–**c** and their protected derivatives **5a**–**c**—see below.

All mentioned compounds were tested at several concentrations. Importantly, the pegylated trehaloses **1a**–**c** were measured at stoichiometric concentrations *w***_1a_** = 18.6%, *w***_1b_** = 5.7% and *w***_1c_** = 5.1% to achieve comparable conditions of cryoprotection. For the same reason, the tested compounds PEG_x_, trehalose, and **1a**–**c** were treated at corresponding molar ratio 2:1:1. We also used effective concentrations *w***_1a_** = 18.6%, *w***_1b_** = 8.6% and *w***_1c_** = 7.7% at the highest viability response for those new materials (Figure 2).

Column chromatography was carried out using Merck Kieselgel 60 (60−100 μm). The purification on a Sephadex resin was performed on LH-20 (Sigma-Aldrich) in dichloromethane. Preparative TLC was carried out on 45 × 18 × 0.4 cm loose-layer silica gel containing a UV indicator (system 1-S1). 5,5′-Diazidotrehalose **3** was prepared by a four-step synthesis from trehalose according to [25]. The preparation of new PEGs **4b**–**c** was adapted from the referenced synthesis of derivative **4a [26]**.

### 3.1. General Procedure for Synthesis of Propargylated PEGs ***4a**–**c***

Potassium *tert*-butoxide (x_100_ = 11.1 mmol, x_1500_ = 0.67 mmol, x_6000_ = 1.42 mmoL) was added to dissolved PEG_x_ (x_100_ = 21.4 mmol, x_1500_ = 1.33 mmoL, x_6000_ = 0.50 mmoL) in dry tetrahydrofuran (20 mL, x_100_ or x_1500_) or dimethylformamide (20 mL, x_6000_, dissolution of PEG by heating) at 0 °C. After 15 min, the mixture was allowed to warm to room temperature and stirred for 30 min. Propargyl bromide was added (x_100_ = 13.4, mmol, x_1500_ = 0.67 mmoL, x_6000_ = 1.98 mmol) and the mixture was stirred overnight for 14 h at the same temperature. The fine solids were filtered off through a membrane filter and the solution was concentrated in vacuo to a minimum volume. In the case of the mixture containing PEG 6000, the reaction was stopped by dilution with dichloromethane (30 mL), the residue was precipitated with diethylether (3 × (5–10) mL), diethylether was decanted and the product was dried on an oil pump.

*2-(2-(prop-2-yn-1-yloxy)ethoxy)ethanol* (**4a**) Yield 2.23 g (91%) of pale yellow liquid. ^1^H NMR (CDCl_3_): δ 2.45 (t, *J =* 2.3, 2H, CH_2_CC**H**), 3.58–3.67 (m, 2H, CH_2_OH), 3.69–3.78 (m, 6H, CH_2_O), 4.22 (d, *J =* 2.4, 2H, C**H**_2_CCH). ^13^C NMR (CDCl_3_): δ 58.6 (**C**H_2_CCH). 61.9, 69.3, 70.4, 72.6 (**C**H_2_O), 74.8 (CH_2_C**C**H), 79.6 (CH_2_**C**CH). The spectroscopic data of **5a** are in agreement with [26].

*2-(2-(prop-2-yn-1-yloxy)polyethylenglycol_1500_* (**4b**) Yield 1.21 g (59%) of white amorphous solid. ^1^H NMR (CDCl_3_): δ 2.44 (t, *J =* 2.4, 2H, CH_2_CC**H**), 3.55–3.72, 3.65 (m, 224H, CH_2_O), 4.20 (d, *J =* 2.3, 2H, C**H**_2_CCH). ^13^C NMR (CDCl_3_): δ 58.4 (**C**H_2_CCH), 61.6, 69.1, 70.2, 70.3, 70.5, 72.6 (**C**H_2_O), 74.6 (CH_2_C**C**H), 79.5 (CH_2_**C**CH). MALDI-TOF: *m*/*z* [M + Na]^+^ calcd for C_69_H_136_O_34_Na, 1508.8; found 1508.7.

*2-(2-(prop-2-yn-1-yloxy)polyethylenglycol_6000_* (**4c**) Yield 2.88 g (95%) of white amorphous solid. ^1^H NMR (CDCl_3_): δ 2.43 (t, *J =* 2.4, 2H, CH_2_CC**H**), 3.54–3.75, 3.63 (m, 663H, CH_2_O), 4.19 (d, *J =* 2.4, 2H, C**H**_2_CCH). ^13^C NMR (CDCl_3_): δ 58.4 (**C**H_2_CCH), 61.5, 69.1, 70.2, 70.4, 72.5 (**C**H_2_O), 74.6 (CH_2_C**C**H), 79.4 (CH_2_**C**CH). MALDI-TOF: *m*/*z* [M + Na]^+^ calcd for C_283_H_564_O_141_Na, 6220.9; found 6220.8.

### 3.2. General Procedure for Synthesis of Pegylated Trehaloses ***5a**–**c***

A mixture of propargylated PEG_x_ (**4a** = 0.86 mmol, **4b** = 0.78 mmol, **4c** = 0.44 mmol) in tetrahydrofuran (10 mL, aqueous solutions of CuSO_4_.5H_2_O (x_100_ = x_1500_ = 0.78 mmol/2 mL, x_6000_ = 0.44 mmol/2 mL) and sodium ascorbate (x_100_ = 86 mmol/2mL, x_1500_ = 0.78 mmol/2 mL, x_6000_ = 0.44 mmol/2 mL) was stirred at room temperature. After 20 min, 5′-azidotrehalose **3** (x_100_ = 86 mmol, x_1500_ = 0.78 mmol, x_6000_ = 0.44 mmol) in tetrahydrofuran (20 mL) was added dropwise. The resulting mixture was stirred for 2–3 h until the conversion of the starting material was complete (monitoring by ^1^H NMR spectroscopy). The mixture was stirred overnight for 14 h at room temperature and then dilluted with tetrahydrofuran (30 mL) and allowed to stand over magnesium sulphate. The mixture was filtered and concentrated in vacuo to a minimum volume. The residue was codistilled with diethylether (10 mL) and decanted with diethylether (3 × 10 mL). The mixture was purified through Sephadex LH-20 in dichloromethane and fractions containing the product were evaporated to dryness. The residue was chromatographed on a preparative TLC (products **5a** and **5b**, S1) or a silica gel column (product **5c**) in dichloromethane/methanol 15:2.

(2*S*,3*S*,4*R*,5*S*)-2-((4-((2-(2-hydroxyethoxy)ethoxy)methyl)-1*H*-1,2,3-triazol-1-yl)methyl)-6-(((3*R*,4*S*,5*R*,6*R*)-3,4,5-tris(benzoyloxy)-6-((4-((2-(2-hydroxyethoxy)ethoxy)methyl)-1*H*-1,2,3-triazol-1-yl)methyl)tetrahydro-2*H*-pyran-2-yl)oxy)tetrahydro-2*H*-pyran-3,4,5-triyl tribenzoate (**5a**). Yield 556 mg (55%) of yellow oil. ^1^H NMR (CDCl_3_): δ 3.60–3.65 (m, 4H, C**H**_2_OH), 3.68–3.78 (m, 12H, CH_2_O), 4.17 (m, 4H, C**H**_2_CHO), 4.25 (m, 2H, OC**H**CH_2_), 4.73 (s, CH_2_C=), 5.23 (t, 2H, *J* = 9.98, CH), 5.34 (dd, 2H, *J =* 9.98, CH), 5.50 (d, 2H, *J =* 4.11, OCHO), 6.19 (2H, dd, *J =* 9.97, *J =* 9.39, CH), 7.27–7.62 (m, 18H, CH= arom), 7.64 (s, 2H, CH=CN), 7.78 (dd, 4H, *J =* 7.0, *J =* 1.2, CH= o-arom.), 7.86 (dd, 4H, *J =* 7.0, *J =* 1.8, CH= o-arom.), 8.09 (dd, 4H, *J =* 7.6, *J =* 1.2, CH= o-arom.). ^13^C NMR (CDCl_3_): δ 61.7 (**C**H_2_CH), 64.6 (**C**H_2_C=), 68.9, 69.0, 69.9, 70.6 (CH), 69.7, 70.4, 72.6 (CH_2_O), 91.7 (CHO), 123.7 (**C**H=CN), 128.26, 128.77 (C= arom.), 128.3, 128.4, 129.9, 129.7, 130.0 (CH= o,m-arom.), 133.3, 133.7, 133.8 (CH= p-arom.), 145.3 (N**C**=CH) 164.9, 165.0, 165.4 (C=O). ESIMS: *m*/*z* 1327 [M + Na]^+^ (72); HRMS (ESI): for C_68_H_68_O_21_N_6_Na (M + Na) calculated, 1327.4; found, 1327.4.

(2*S*,3*S*,4*R*,5*S*)-2-((4-((polyethylenglycol_1500_-yl)oxy)methyl)-1H-1,2,3-triazol-1-yl)methyl)-6-(((3*R*,4*S*,5*R*,6*R*)-3,4,5-tris(benzoyloxy)-6-((4-((polyethylenglycol_1500_-yl)oxy)methyl)-1*H*-1,2,3-triazol-1-yl)methyl)tetrahydro-2*H*-pyran-2-yl)oxy)tetrahydro-2*H*-pyran-3,4,5-triyl tribenzoate (**5b**). Yield 388 mg (13%) of yellow melting amorphous solid. ^1^H NMR (CDCl_3_): δ 3.55–3.75 (m, 337H, C**H**_2_O), 4.10 (m, 4H, C**H**_2_CHO), 4.31 (m, 2H, OC**H**CH_2_), 4.69 (s, 4H, CH_2_C=), 5.19 (t, 2H, *J =* 9.98, CH), 5.30 (dd, 2H, *J =* 4.06, CH), 5.51 (d, 2H, *J =* 3.53, OCHO), 6.19 (2H, dd, *J =* 9.97, *J =* 9.37, CH), 7.28–7.61 (m, 30H, CH= arom), 7.51 (s, 2H, CH=CN), 7.78 (dd, 2H, *J =* 7.0, *J =* 1.8, CH= o-arom.), 7.86 (dd, 4H, *J =* 7.6, *J =* 1.2, CH= o-arom.), 8.10 (dd, 4H, *J =* 7.0, *J =* 1.2, CH= o-arom.). ^13^C NMR (CDCl_3_): δ 61.6 (**C**H_2_CH), 65.9 (**C**H_2_C=), 68.8, 68.9, 69.0, 70.9 (CH), 69.4, 69.7, 70.1, 70.5, 72.6 (CH_2_O), 91.7 (CHO), 123.7 (**C**H=CN), 128.30, 128.77 (C= arom.), 128.3, 128.36, 128.41, 128.8, 129.8, 129.9, 130.0 (CH= o,m-arom.), 133.3, 133.7, 133.8 (CH= p-arom.), 145.3 (N**C**=CH) 164.8, 165.0, 165.4 (C=O). MALDI-TOF: *m*/*z* [M + Na]^+^ calcd for C_192_H_316_O_83_N_6_Na, 4059.3; found 4059.5.

(2*S*,3*S*,4*R*,5*S*)-2-((4-((polyethylenglycol_6000_-yl)oxy)methyl)-1*H*-1,2,3-triazol-1-yl)methyl)-6-(((3*R*,4*S*,5*R*,6*R*)-3,4,5-tris(benzoyloxy)-6-((4-((polyethylenglycol_6000_-yl)oxy)methyl)-1*H*-1,2,3-triazol-1-yl)methyl)tetrahydro-2*H*-pyran-2-yl)oxy)tetrahydro-2*H*-pyran-3,4,5-triyl tribenzoate (**5c**) Yield 967 mg (29%) of yellowish melting amorphous solid. ^1^H NMR (CDCl_3_): δ 3.37–3.87 (m, 1416H, C**H**_2_O), 4.10 (m, 4H, C**H**_2_CHO), 4.30 (m, 2H, OC**H**CH_2_), 4.69 (s, 4H, CH_2_C=), 5.18 (t, 2H, *J =* 9.98, CH), 5.30 (dd, 2H, *J =* 3.52, CH), 5.48 (d, 2H, *J =* 3.52, OCHO), 6.17 (2H, dd, *J =* 9.98, *J =* 9.39, CH), 7.28–7.61 (m, 30H, CH= arom), 7.51 (s, 2H, CH=CN), 7.76 (dd, 2H, *J =* 7.0, *J =* 1.2, CH= o-arom.), 7.85 (dd, 4H, *J =* 7.0, *J =* 1.2, CH= o-arom.), 8.08 (dd, 4H, *J =* 7.0, *J =* 1.2, CH= o-arom.). ^13^C NMR (CDCl_3_): δ 61.7 (**C**H_2_CH), 66.8 (**C**H_2_C=), 68.8, 69.0, 69.9, 70.9 (CH), 68.8–72.7 (CH_2_O), 91.9 (CHO), 123.7 (**C**H=CN), 128.28, 128.79 (C= arom.), 128.3, 128.4, 128.8, 129.0, 129.8, 129.9, 130.0 (CH= o,m-arom.), 133.3, 133.7, 133.9 (CH= p-arom.), 145.3 (N**C**=CH) 164.8, 165.0, 165.4 (C=O). MALDI-TOF: *m*/*z* [M + Na]^+^ calcd for C_620_H_1181.4_O_297_N_6_Na, 13486.6; found 13486.6.

### 3.3. General Procedure for Synthesis of Deprotected PEG-Hybrids ***1a**–**c***

A mixture of pegylated trehalose (**5a** = 0.48 mmol, **5b** = 0.15 mmol, **5c** = 0.056 mmol) and sodium methoxide (x_100_ = 0.54 mmol, x_1500_ = 0.22 mmol, x_6000_ = 0.13 mmol) in methanol (10–30 mL) was stirred overnight for 14 h at room temperature. The mixture was neutralized by treatment with Dowex 50 (H^+^ form) and filtrated. The solution was concentrated in vacuo to a minimum volume. The mixture was distilled with hexane (2 × 2 mL), extracted and decanted with hexane (3 × 10 mL) and ether (3 × 10 mL). The residue containing **1a** or **1b** was chromatographed on a preparative TLC (S1) in dichloromethane (*R*_f_ = 0). The residue containing **1c** was purified on Sephadex LH-20 in dichloromethane. The product was evaporated in vacuo to dryness, distilled and triturated with diethylether (10 mL). The product was dried on an oil pump.

(2*S*,3*R*,4*R*,5*S*)-2-((4-((2-(2-hydroxyethoxy)ethoxy)methyl)-1*H*-1,2,3-triazol-1-yl)methyl)-6-(((3*R*,4*S*,5*S*,6*R*)-3,4,5-trihydroxy-6-((4-((2-(2-hydroxyethoxy)ethoxy)methyl)-1*H*-1,2,3-triazol-1-yl)methyl)tetrahydro-2H-pyran-2-yl)oxy)tetrahydro-2H-pyran-3,4,5-triol (**1a**). Yield 212 mg (77%) of white foam. ^1^H NMR (DMSO): δ 2.91 (m, 2H, *J =* 9.1, *J =* 5.28, CH_2_O**H**), 3.31–3.58 (m, 16H, CH_2_O), 4.09 (m, 6H, CHO**H**), 4.44 (m, 2H, *J =* 7.04, *J =* 7.63, C**H**CH_2_), 4.48 (s, 4H, C**H**_2_C=), 4.52 (m, 4H, CHC**H**_2_), 4.59 (dd, 2H, *J =* 5.87, *J =* 5.28, CH), 4.90 (d, 2H, CH, *J =* 5.87), 4.97, 5.31 (2x d, 2H, CH, *J =* 5.28), 7.93 (s, 2H, CH=). ^13^C NMR (DMSO): δ 60.1 (**C**H_2_CH), 63.3 (**C**H_2_C=), 68.8, 69.7, 72.4 (CH_2_O), 69.8, 71.2, 72.6, 93.7 (CHO), 124.5 (**C**H=CN), 143.7 (N**C**=CH). ESIMS: *m*/*z* 703 [M + Na]^+^ (86); HRMS (ESI): for C_26_H_44_O_15_N_6_Na [M + Na]^+^ calculated, 703.29; found, 703.29.

(2*S*,3*R*,4*R*,5*S*)-2-((4-((polyethylenglycol_1500_-yl)oxy)methyl)-1*H*-1,2,3-triazol-1-yl)methyl)-6-(((3*R*,4*S*,5*S*,6*R*)-3,4,5-trihydroxy-6-((4-((polyethylenglycol_1500_-yl)oxy)methyl)-1*H*-1,2,3-triazol-1-yl)methyl)tetrahydro-2*H*-pyran-2-yl)oxy)tetrahydro-2*H*-pyran-3,4,5-triol (**1b**). Yield 188 mg (63%) of yellowish amorphous solid. ^1^H NMR (DMSO): δ 2.91 (m, 2H, *J =* 7.04, *J =* 5.28, CH_2_O**H**), 3.06–3.76 (m, 268H, CH_2_O), 4.08 (m, 6H, CHO**H**), 4.39 (m, 2H, *J =* 7.05, *J =* 7.63, C**H**CH_2_), 4.48 (s, 4H, C**H**_2_C=), 4.52 (m, 4H, CHC**H**_2_), 4.58 (dd, 2H, *J =* 5.87, *J =* 5.28, CH), 4.91 (d, 2H, CH, *J =* 5.87), 4.97, 5.31 (2x d, 2H, CH, *J =* 5.28), 7.92 (s, 2H, CH=). ^13^C NMR (DMSO): δ 60.2 (**C**H_2_CH), 63.3 (**C**H_2_C=), 68.7, 69.8, 72.3 (CH_2_O), 71.2, 72.6, 93.8 (CHO), 124.4 (**C**H=CN), 143.7 (N**C**=CH). MALDI-TOF: *m*/*z* [M + Na]^+^ calcd for C_150_H_290_O_77_N_6_Na, 3388.7; found 3388.9.

(2*S*,3*R*,4*R*,5*S*)-2-((4-((polyethylenglycol_6000_-yl)oxy)methyl)-1*H*-1,2,3-triazol-1-yl)methyl)-6-(((3*R*,4*S*,5*S*,6*R*)-3,4,5-trihydroxy-6-((4-((polyethylenglycol_6000_-yl)oxy)methyl)-1*H*-1,2,3-triazol-1-yl)methyl)tetrahydro-2*H*-pyran-2-yl)oxy)tetrahydro-2*H*-pyran-3,4,5-triol (**1c**). Yield 309 mg (36%) of yellowish amorphous solid. ^1^H NMR (DMSO): δ 2.91 (m, 2H, *J =* 9.4, *J =* 5.28, CH_2_O**H**), 3.19–3.78 (m, 1075H, CH_2_O), 4.09 (m, 6H, CHO**H**), 4.41 (m, 2H, *J =* 7.05, *J =* 8.22, C**H**CH_2_), 4.48 (s, 4H, C**H**_2_C=), 4.52 (m, 4H, CHC**H**_2_), 4.59 (t, 2H, *J =* 5.28, CH), 4.90 (d, 2H, CH, *J =* 5.87), 4.97 (d, 2H, CH, *J =* 4.70), 5.31 (d, 2H, CH, *J =* 5.28) 7.92 (s, 2H, CH=). ^13^C NMR (DMSO): δ 60.2 (**C**H_2_CH), 63.3 (**C**H_2_C=), 68.7, 69.8, 72.4 (CH_2_O), 71.2, 72.6, 93.8 (CHO), 124.4 (**C**H=CN), 143.7 (N**C**=CH). MALDI-TOF: *m*/*z* [M + Na]^+^ calcd for C_578_H_1150_O_291_N_6_Na, 12813.8; found 12813.5.

### 3.4. Cell Treatment

The cells used in experiments were Z138-MCL human mantle cell lymphoma (detailed characteristics available at http://www.lgcstandards-atcc.org/Products/All/CRL-3001.aspx?geo_country=cz). The cells were cultivated at 37 °C in a humidified atmosphere with 5% CO_2_ in cultivation flasks containing IMDM medium supplemented with 15% FBS and 1% penicillin/streptomycin. We presume that the cryoprotective effects do not significantly rely on the exact cell type used (unless it is a very special cell type such as sperm cells or oocytes) as standard cryoprotectants such as DMSO are also used for preservation of any common cell type. The differences between cryoprotective agents manifest on any standard type of somatic cells.

#### 3.4.1. Cell Freezing

The cells were put on a 96-well plate, with each well containing about 5 × 10^5^ cells. The plate was then centrifuged (460 g, 2 min) and after removal of the supernatant, the plate was shortly vortexed. 200 µL of respective cryoprotectant solution was added to each well, and the plate was incubated for 20 min at room temperature. Then, the plate was put in a freezer at −80°C for 24 h.

#### 3.4.2. Cell Thawing

Samples were thawed by leaving the plate in a 37 °C incubator until the ice melted (approx. 30 min). The plate was then certifuged, the supernatant was discarded, the plate was vortexed, and cells were washed twice in Annexin-binding buffer (Invitrogen-Thermo Fisher Scientific).

### 3.5. Flow Cytometry

#### Staining

To each well, 10 µL of the annex. buffer containing 0.5 µL of annexin-V/APC (ThermoFisher Scientific https://www.thermofisher.com/order/catalog/product/A35110) was added, and the plate was incubated for 15 min at room temperature. After that, propidium iodide was added to each well (10 µL of c = 1 µg/mL) and the plate was immediately taken to be measured by flow cytometer. The cytometry measurement began 30–45 min after the thawing. To evaluate the cryoprotective efficiency of tested cryoprotectants, we measured live cell count (LCC). This viability parameter represents the number of detected live cells (AnnexinV–PI–, i.e., non-apoptotic and non-necrotic), with debris and multiplets excluded. Determining the LCC was possible due to measuring equal volumes (15 µL) for each sample. LCC is a better characteristic of cryoprotectant efficiency than relative survival (fraction of live cells in all recorded cells). This is because a variable number of dead cells are fragmented during freezing and are not detected in the measurement.

For the detection of cell viability, we used the standard method of fluorescent cell staining by Propidium iodide (PI) and fluorescent-labeled Annexin V, and their detection by flow cytometry. Propidium iodide is a fluorescent DNA-intercalating agent that stains DNA. As it is not membrane-permeable, it can only enter cells with disrupted membrane integrity—both apoptotic and necrotic cells. To distinguish apoptotic from necrotic cells, we used fluorescent-labeled Annexin V. Annexin V is a protein that specifically binds to phosphatidylserine (PS)—a phospholipid component of the cell membrane. As PS molecules are only located in the inner leaflet of the cell membrane, they are not accessible for staining in living cells. However, during apoptosis, PS is being translocated to the outer leaflet, making it available for binding of Annexin V. Measuring the fluorescence of the two stains by flow cytometry enables us to distinguish 4 distinct subsets in the total cell population:

- Annexin V-positive, PI-negative: early apoptotic cells (PS translocation took place, but membrane integrity is still intact)
- Annexin V-positive, PI-positive: late apoptotic cells (PS translocation took place and the membrane is permeable for PI)
- Annexin V-negative, PI-positive: necrotic cells (PS translocation did not take place, but the membrane is disrupted and permeable)
- Annexin V-negative, PI-negative: live cells (no PS translocation and membrane is intact).

To show the live cells, we used cell counts from the last, double-negative, population.

## 4. Conclusions

In this work, we described a simple click-condensation of propargylated PEGs 100, 1500 and 6000 with trehalose 5,5′-diazidoderivative **3** as a new efficient synthetic method for polymer insertion into the structure of trehalose as natural cryoprotectant. This new chemical compound appears to be a relevant way to show the PEG and trehalose functions in cryoprotectective process. Based on the potential dynamic change of cryoprotectant behavior near cell membranes, we outlined that trehalose chemically bound to extracellular polymeric cryoprotectants could be helpful as a chemical tool for the evaluation of polymeric cryoprotectants cell membrane influences. These effects are still not easily predicted and understood. Lower cryoprotective effect of new products related to a mixture of PEG and trehalose could be explained by the specific interaction of trehalose and PEG moiety with the cell membranes. Typically, water on the cell surface is replaced by protective trehalose. Short PEGs (100) with high probability remove the cell surface hydration layer which destabilizes cell membrane (low cryoprotective effect). Longer PEG chains (PEG 1500, PEG 6000) do not so strongly (as PEG 100) destabilize the cell surface and work as cryoprotectant mainly inhibiting ice crystal growth (higher cryoprotective effect)—long PEGs may have more conformations that prevent them from sticking more closely to the membranes than the shorter PEGs. If long PEG and trehalose are mixed, water on the surface is replaced by protective trehalose and long PEG chains inhibit ice crystal growth and due to the fact that PEGs are not close to the cell surface do not importantly destabilize cell membrane.

If PEG and trehalose are chemically bound (pegylated hybrids **1a**-**1c)**, trehalose is adsorbed on the cell surface and PEGs molecules which are due to the chemical bonding with trehalose close to the cell surface remove the cell surface hydration layer and destabilize the cell membrane which was confirmed by the fact that cryoprotective effect of pegylated hybrid **1a** (containing PEG 100) > cryoprotective effect of pegylated hybrid **1c** (containing PEG 6000) > cryoprotective effect of pegylated hybrid **1b** (containing PEG 1500). The cryoprotective effect of pegylated hybrid **1a** suggests a marginal role of short PEG chains destabilizing interactions with water on the cell surface. While the low “chelate” effect of pegylated hybrid is in agreement with measurements for short alkyl chains, long tentacular pegylated chains can be more flexible to form tight hydrogen interactions.

In the future we would like to reveal the polymer interactions with membrane using infrared spectroscopy. For these purposes, the combination of DPPC and charged DMPC or POPC membranes could be used. Differences of infrared spectra may provide valuable results of water-coating behavior toward the membrane phospholipids in varying temperatures. Especially, the IR spectroscopy measurements of our hybrid compounds, resulting from the connection of dehydrating and osmolytic properties of PEG and trehalose, would be essential for confirmation of the reported proposals of their function.

Modification of membrane interactions through replacement of water with cryoprotectant hybrids could reveal assumed changes of osmotic processes as an important cryoprotective factor, where membrane stability and cryoprotection is discussed, however, also not fully evaluated with respect to cell-specific effects. All these unexplored cryoprotective factors, as well as the development of new syntheses of other useful polyfunctional compounds derived from sugar-modified polymers, are included in the topics of our future research.

## Supplementary Materials

The following are available online, Scheme S1. Direct condensation of PEG-polymer to 5‘-bromo-substituted trehalose synthon [25]. Figure S1: ^1^H NMR and ^13^C NMR spectra copies.
