# Supplementary material for: Simple Syntheses of New Pegylated Trehalose Derivatives as a Chemical Tool for Potential Evaluation of Cryoprotectant Effects on Cell Membrane
Source: Molecules. 2020 Jan 23;25(3):497. doi: 10.3390/molecules25030497 (PMC7038055; doi:10.3390/molecules25030497)
Supplement: Supplementary file 1 [file molecules-25-00497-s001.pdf]

Simple syntheses of new pegylated trehalose derivatives as a chemical tool for evaluation of cryoprotectant effects on cell membrane

Karel Pomeisl, Martin Golan, Jan Richter and Irena Kratochvílová*

Institute of Physics, Czech Academy of Sciences, Na Slovance 1999/2, 182 21 Praha 8, Czech Republic

*Corresponding author: krat@fzu.cz
Keywords: click-chemistry, pegylation, trehalose, PEG, cryoprotection, membrane stability, chelate effect

The direct alkylation of 6′-bromoderivitave of trehalose (9)

The low reactivity, selectivity and specific methods of disacharide skeleton protection lead us to symmetrical modification at 5'-carbon to activate a potential polymer spacer. The polymer insertion at the 5'-carbon also seems to be more appropriate for spatial direction of pegylated tentacles while secondary hydroxyl groups are still available for assumed membrane interactions. For that reason, we first focused on the simple modification of the 5‘-hydroxyl group by the substitution of activated trehalose intermediate **8** with bromine using *N*-bromosuccinimide [25] to give 5‘-bromoderivative **9** (see Scheme S1).

The intermediate **8** was obtained by a two-step synthesis from trehalose **2** through the protected intermediate **7** according to [25]. Unfortunately, the direct condensation of **9** with PEG 1500 in the presence of various bases and under various reaction condition did not afford the corresponding polymer hybrid **5** (the reaction mixtures were monitored by NMR spectroscopy).

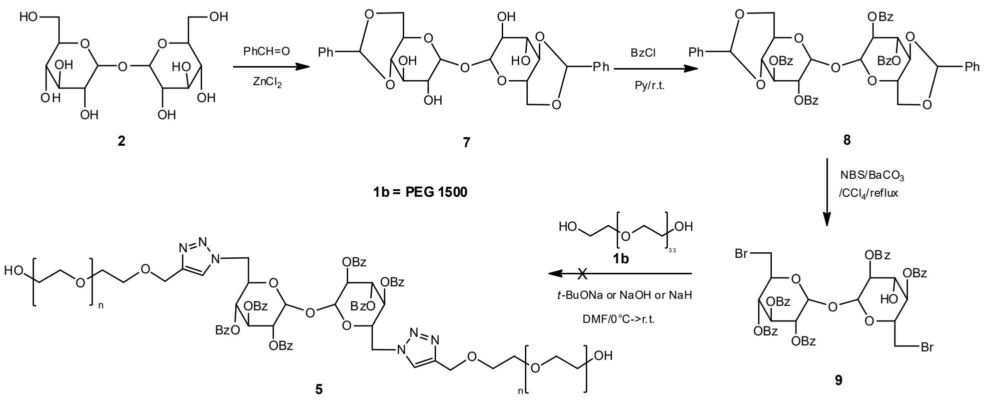

Scheme S1. Direct condensation of PEG-polymer to 5'-bromo-substituted trehalose synthon [25] .

Fig. S1: ^{1}H NMR and ^{13}C NMR spectra copies

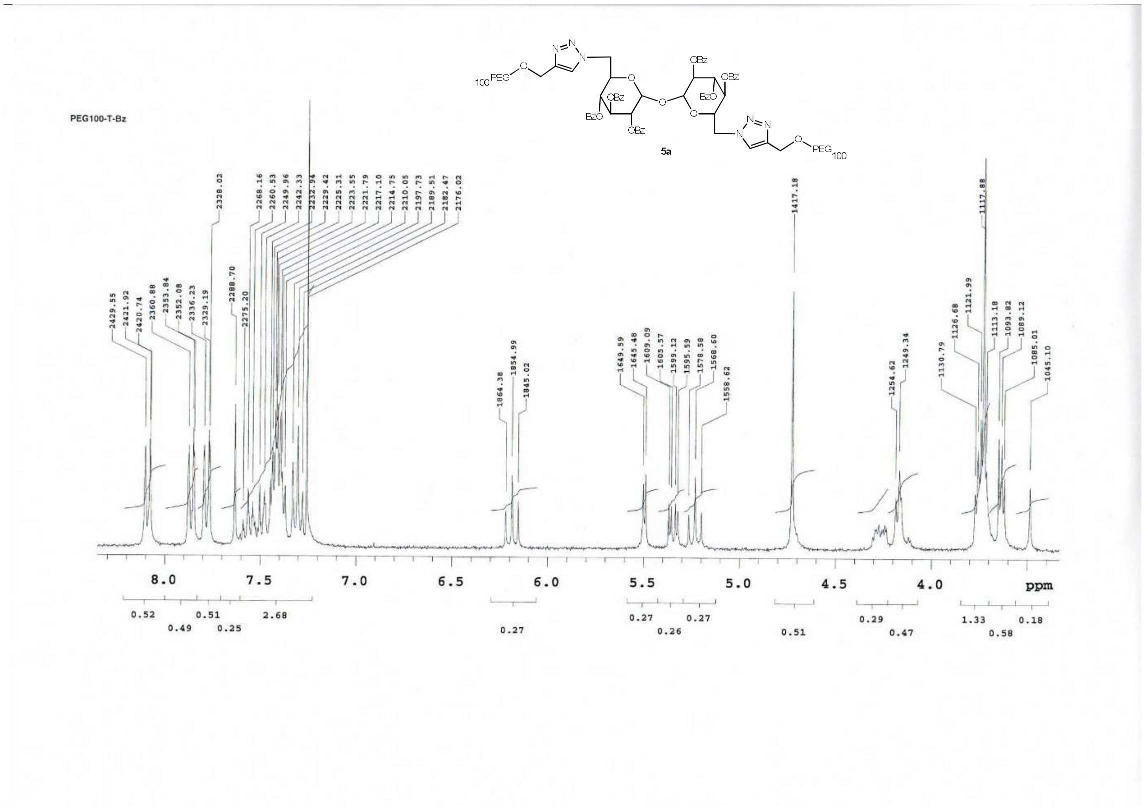

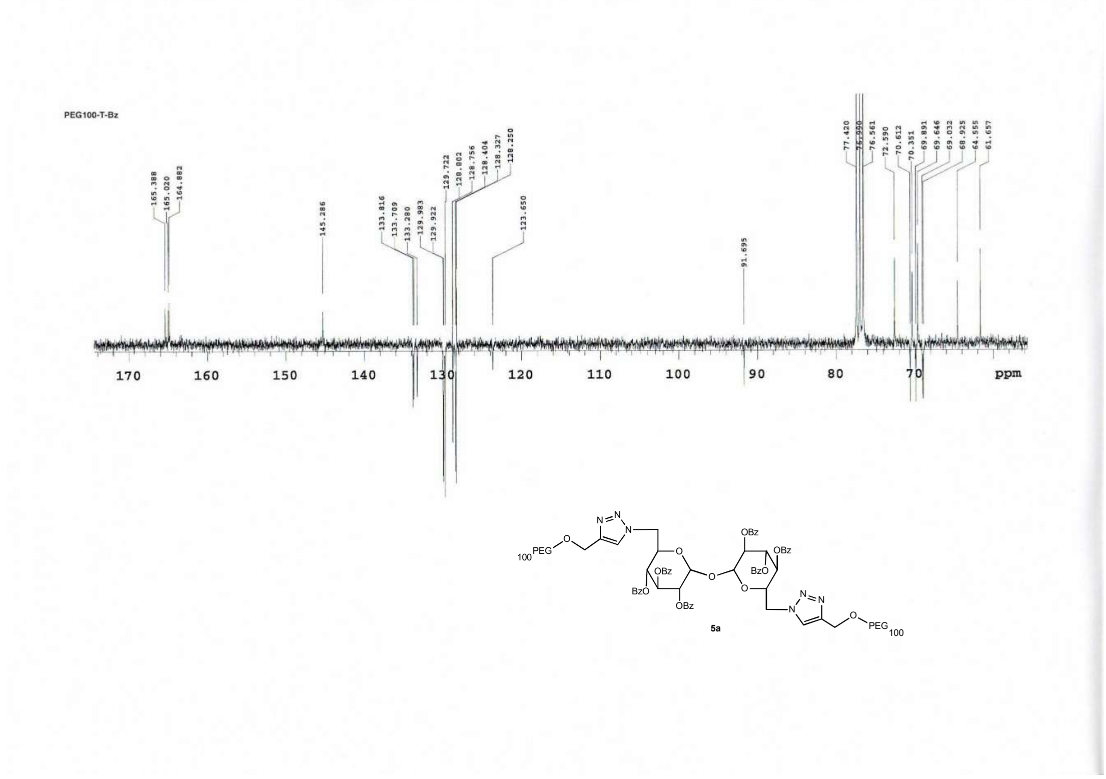
PEG100-T-Bz
165.388
165.020
164.882
145.286
133.816
133.709
133.280
129.983
129.922
129.722
128.802
128.756
128.404
128.327
128.250
123.650
91.695
77.420
76.920
76.561
72.590
70.612
70.351
69.891
69.646
69.032
68.925
64.555
61.657
170
160
150
140
130
120
110
100
90
80
70
ppm
100PEG
OBz
BzO
5a
PEG100

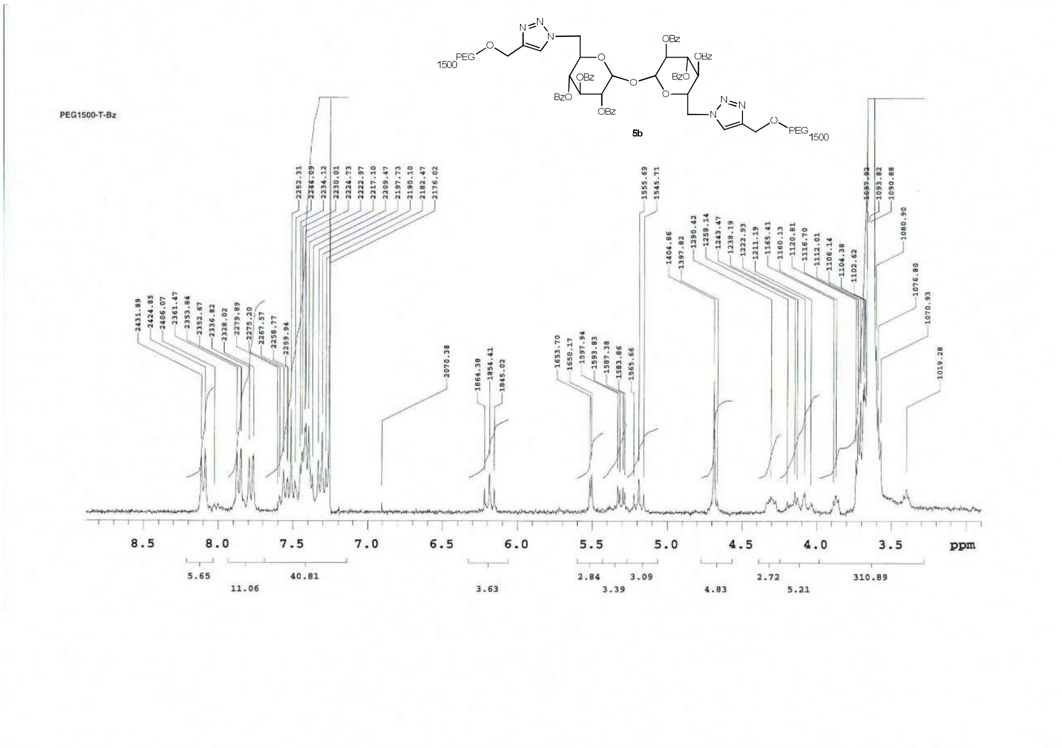
PEG1500-T-Bz
1500PEG
OBz
BzO
5b
PEG1500
8.5
8.0
7.5
7.0
6.5
6.0
5.5
5.0
4.5
4.0
3.5
ppm
5.65
11.06
40.81
3.63
2.84
3.39
3.09
4.83
2.72
5.21
310.89

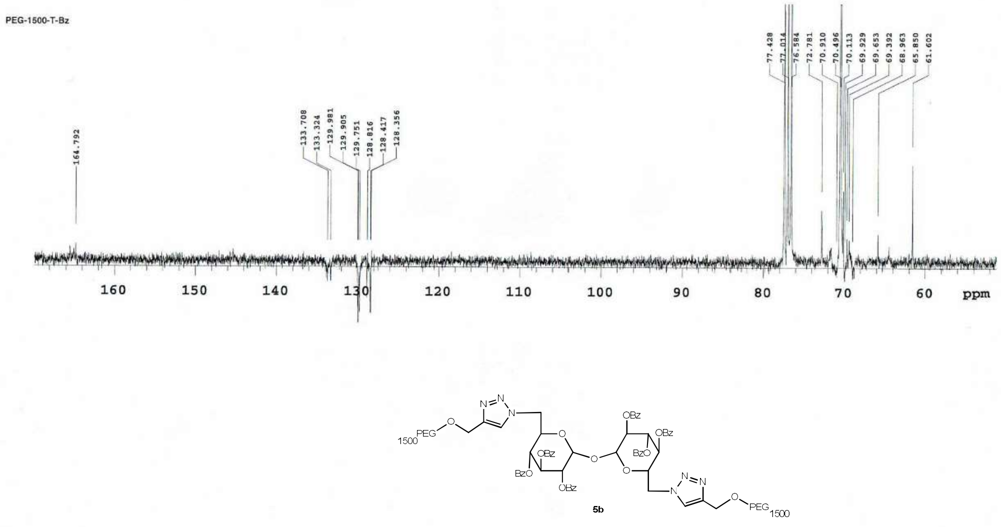
PEG-1500-T-Bz
164.792
133.708
133.324
129.981
129.905
129.751
128.816
128.417
128.356
77.428
77.014
76.584
72.781
70.910
70.496
70.113
69.929
69.653
69.392
68.963
65.850
61.602
160 150 140 130 120 110 100 90 80 70 60 ppm
1500 PEG
OBz
BzO
5b
PEG 1500

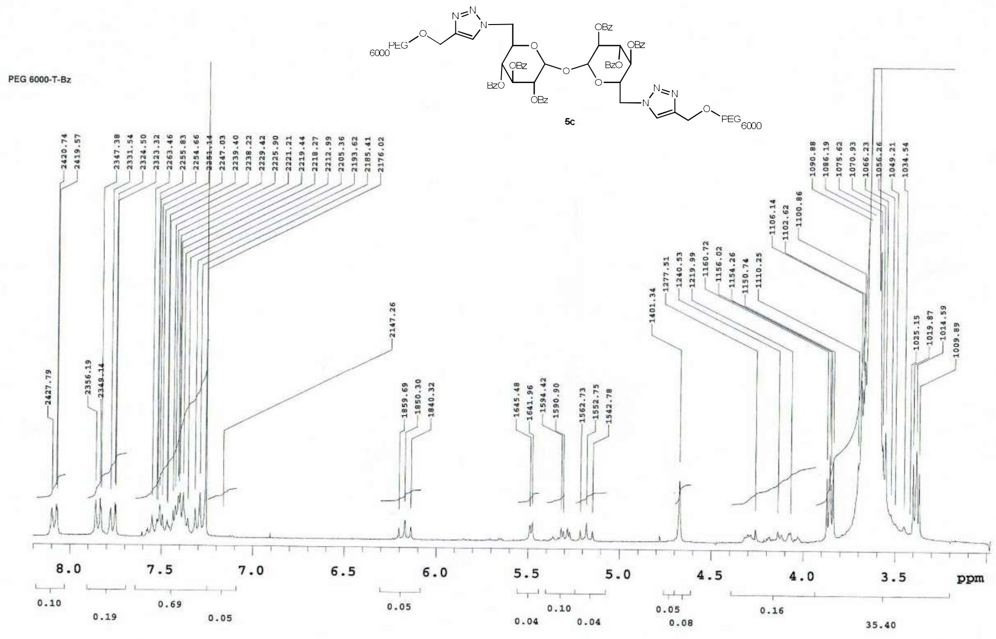
PEG 6000-T-Bz
6000 PEG
OBz
BzO
5c
PEG 6000
2427.79
2420.74
2419.57
2356.19
2349.14
2347.38
2331.54
2324.50
2147.26
1859.69
1850.30
1840.32
1645.48
1641.96
1594.42
1590.30
1562.73
1552.75
1542.78
1401.34
1277.51
1240.53
1219.99
1160.72
1156.02
1154.26
1150.74
1110.25
1106.14
1102.62
1100.86
1090.88
1086.19
1075.62
1070.93
1066.23
1056.26
1049.21
1034.54
1025.15
1019.87
1014.59
1009.89
8.0 7.5 7.0 6.5 6.0 5.5 5.0 4.5 4.0 3.5 ppm
0.10
0.19
0.69
0.05
0.05
0.04
0.10
0.04
0.05
0.08
0.16
35.40

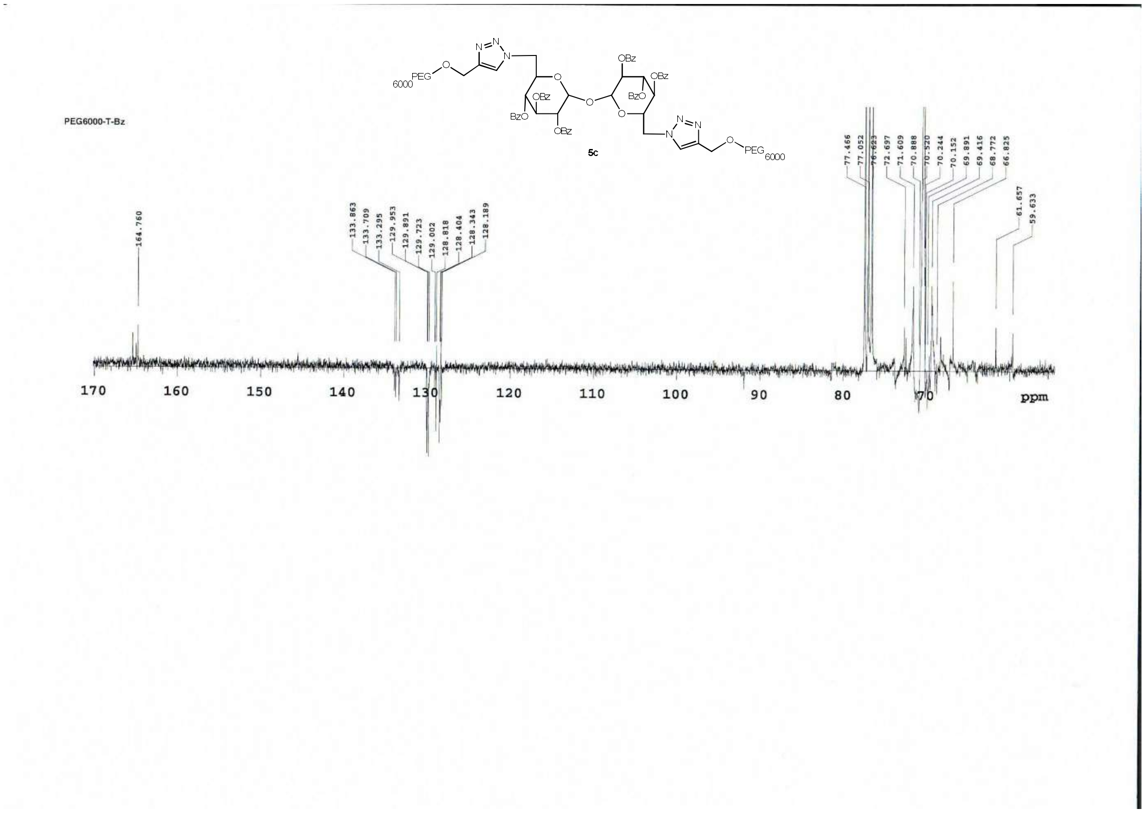
PEG6000-T-Bz
5c
ppm

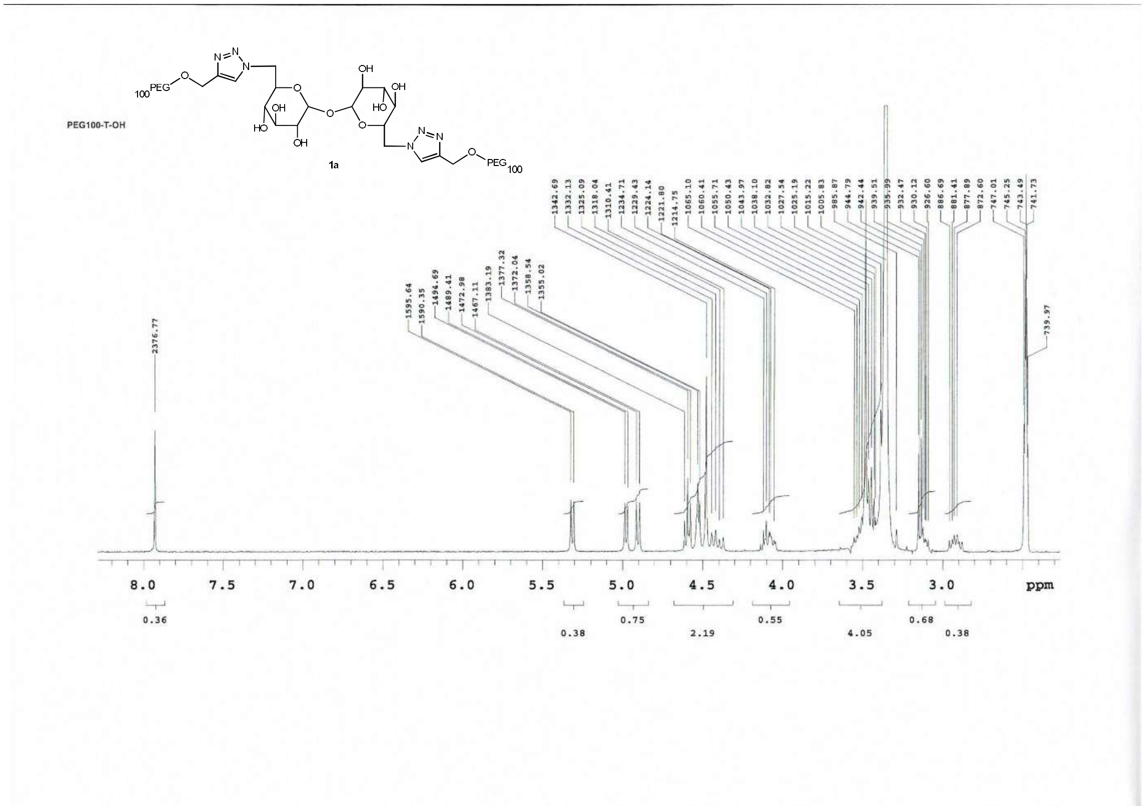
PEG100-T-OH
1a
ppm

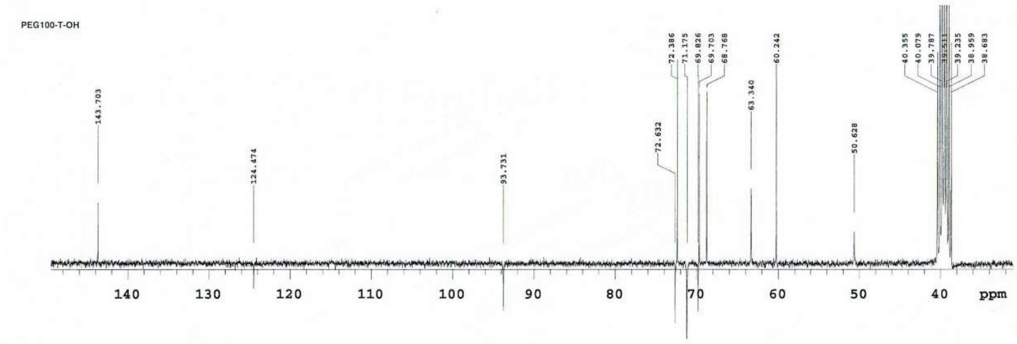
PEG100-T-OH
143.703
124.474
93.731
72.386
71.375
69.826
69.703
68.768
72.632
63.340
60.242
50.628
40.355
40.079
39.787
39.511
39.235
38.959
38.683
140
130
120
110
100
90
80
70
60
50
40
ppm

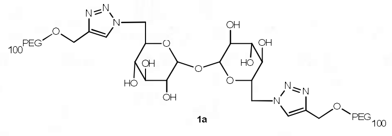
100PEG
O
N=N
N
OH
O
O
HO
OH
HO
OH
OH
O
PEG100

1a

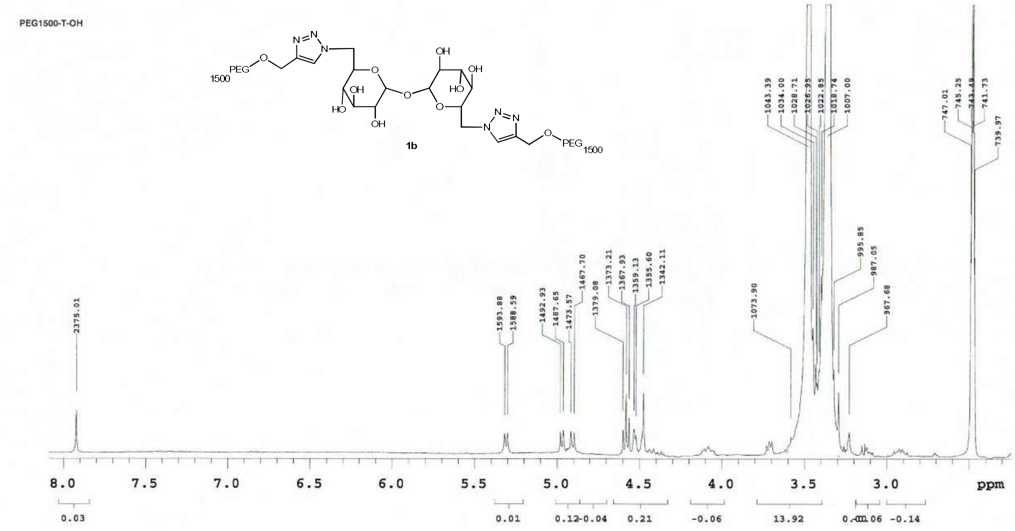
PEG1500-T-OH
1500PEG
PEG1500
1b
2375.01
1593.88
1588.59
1492.93
1487.65
1473.57
1467.70
1379.08
1373.21
1367.93
1359.13
1355.60
1342.11
1073.90
1043.39
1034.00
1028.71
1026.95
1022.85
1018.74
1007.00
995.85
987.05
967.68
747.01
745.25
741.73
739.97
8.0
7.5
7.0
6.5
6.0
5.5
5.0
4.5
4.0
3.5
3.0
ppm
0.03
0.01
0.12
-0.04
0.21
-0.06
13.92
0.06
-0.14

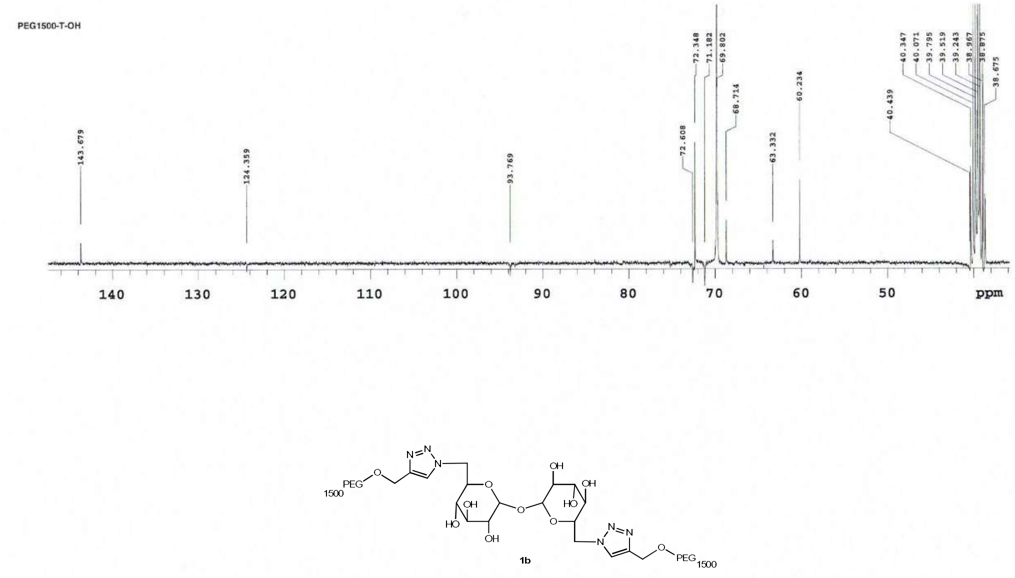
PEG1500-T-OH
143.679
124.359
93.769
72.608
72.348
71.182
69.802
68.714
63.332
60.234
40.439
40.347
40.071
39.795
39.519
39.243
38.967
38.875
38.675
140
130
120
110
100
90
80
70
60
50
ppm
PEG
1500
PEG
1500
1b

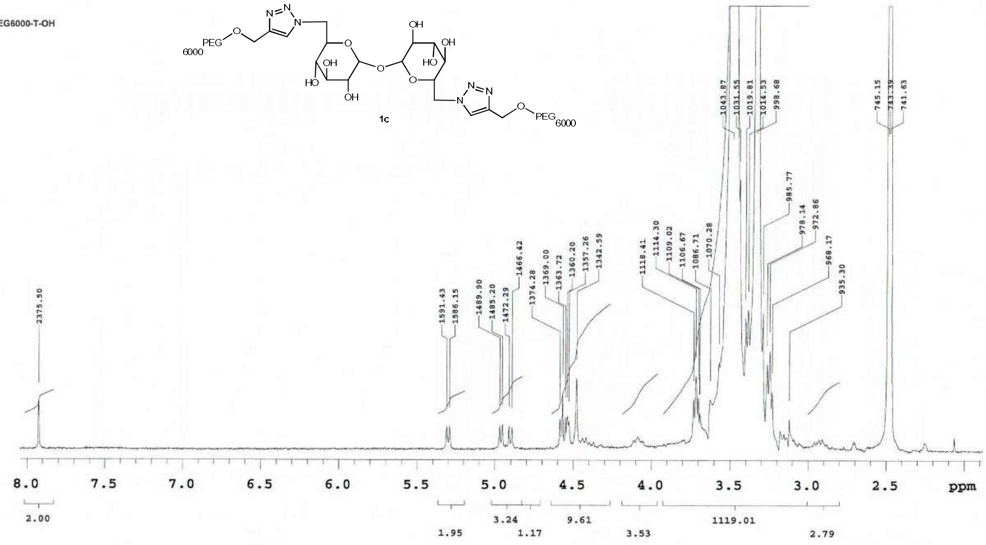
PEG6000-T-OH
PEG
6000
PEG
6000
1c
2375.50
1591.43
1586.15
1489.90
1485.20
1472.29
1466.42
1374.28
1369.00
1363.72
1360.20
1357.26
1342.59
1118.41
1114.30
1109.02
1106.67
1086.71
1070.28
1043.87
1031.55
1019.81
1014.53
998.68
985.77
978.14
972.86
968.17
935.30
745.15
743.39
741.63
8.0
7.5
7.0
6.5
6.0
5.5
5.0
4.5
4.0
3.5
3.0
2.5
ppm
2.00
1.95
3.24
1.17
9.61
3.53
1119.01
2.79

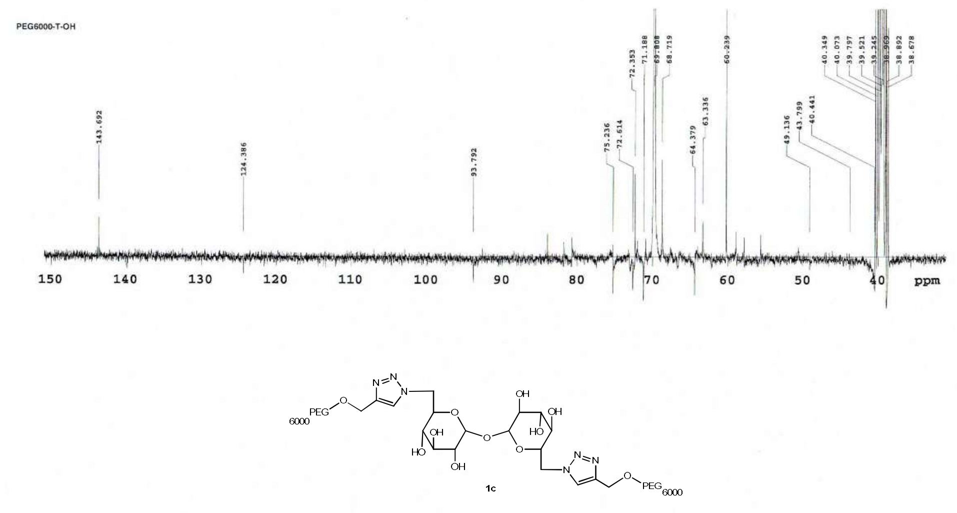
PEG6000-T-OH
143.692
124.386
93.792
75.236
72.614
72.353
71.188
69.808
68.719
64.379
63.336
60.239
49.136
43.799
40.441
40.349
40.073
39.797
39.521
39.245
38.969
38.892
38.678
150
140
130
120
110
100
90
80
70
60
50
40
ppm
PEG
6000
OH
HO
PEG
6000
1c
